# Electrospun Materials for Biomedical Applications

**DOI:** 10.3390/pharmaceutics14081556

**Published:** 2022-07-26

**Authors:** Hernane S. Barud, Frederico B. De Sousa

**Affiliations:** 1 Biopolymers and Biomaterials Laboratory (BioPolMat), University of Araraquara (UNIARA), Araraquara 14801-320, SP, Brazil; 2 Laboratório de Sistemas Poliméricos e Supramoleculares (LSPS), Instituto de Física e Química, Universidade Federal de Itajubá (UNIFEI), Itajubá 37500-903, MG, Brazil

Considered a simple and versatile technique, electrospinning has emerged as a technology for developing 3D materials for a wide range of applications. Electrospun fibers ranging from nanometers to micrometers in diameter have been applied in many research areas including chemistry, materials science, engineering, and chemical engineering, as well as medicine, pharmacology, and pharmaceutics. Based on a large variety of polymers (natural or synthetic ones), types of collectors, and nozzle configurations, a vast and complex number of electrospun materials can be obtained, allowing those electrospun fibers to be applied for catalysis [1], separation and purification [2,3], chemical sensing [4,5], food packaging [6], tissue engineering [7], wound dressing [8], and drug delivery [9], among others. The ability to produce nanofibers with different biocompatible and biodegradable polymers, with low cost, unique physicochemical properties, small diameter, high volume to surface area ratio, has raised interest in the technique for the production of materials applied in drug biomedical applications. These uses can be highlighted as the most prominent ones, and these reviews can provide perspective on the fundamentals of electrospinning for biomedical applications [10,11,12,13,14], among other ones. Figure 1 depicts the number of publications per year, using three sets of keywords (searched within article title, abstract, keywords) using Scopus database: (i) electrospinning, (ii) electrospinning and biomedical, and (iii) electrospinning and drug delivery.

Based on Figure 1, an increasing in the number of publications from 1900–2000 is observed; these publications share the keyword electrospinning. This impact in the electrospinning research area has been related to the activities of the Reneker’s group [15]. The first studies dealing with the uses of electrospun mats for biomedical applications are from 1978 by Annis et al. [16] and 1985 by Fisher et al. [17]. Moreover, in 2021 (highest number of records) the number of documents related to biomedical applications covers about 48%, while those associated to the drug delivery comprises 38% of the total documents. Thus, the technique became a toolbox to design systems of interest, controlling mandatory factors in the drug release process, such as intumescence rate, polymer/drug affinity, polymer degradation rate, and polymer polarity affinity [18,19]. In this Special Issue, great examples of biomedical applications of electrospun fibers are described by articles and reviews.

In their review entitled “Tunable Spun Fiber Constructs in Biomedicine: Influence of Processing Parameters in the Fibers’ Architecture”, Felgueiras et al. described how processing parameters can affect the architecture of electrospun fibers [20]. In addition to the electrospinning principles, the authors have highlighted some important fibers structures, core-sheath (including tri-axial), hollow, porous, side-by-side, and multilayered ones for the biomedical application. The wet-spinning principles and setup were also presented as an important method for obtaining electrospun fibers for tissue engineering and drug delivery applications.

St. John et al., in their review entitled “Advances in Electrospun Nerve Guidance Conduits for Engineering Neural Regeneration”, reported the capability of the electrospun approach to fabricate fibers of various scales and which have a large surface area with a three-dimensional porous structure resembling the native extracellular matrix for regeneration of the nervous system [21]. Considerations regarding conditions of the injury, as well as the requirements and structure for the nerve guidance conduits, were also discussed. This review also reported recent systems focusing on the electrospun uses for regenerating peripheral nerves, demonstrating the vast number of polymers tested. Moreover, the nerve guidance conduits composition and properties were presented, as well as the tissue-stimulating agents. Finally, the authors brought some nerve guidance conduits that are in clinical trials.

In the third review published in this Special Issue—“Osteochondral Tissue Engineering: The Potential of Electrospinning and Additive Manufacturing” by Costa et al.—the most recent advances in osteochondral tissue engineering are summarized [22]. The authors provided an introduction about those challenges related to the osteochondral damage and the advantages and disadvantages of the current therapies. A section dealing with biomaterials for osteochondral tissue engineering construction and the incorporation of biochemical stimuli to improve the tissue response, integration and repair was detailed. Then, the strategy using electrospinning as a building block method for scaffolds preparation and characterization was presented, bringing examples of post-fabrication strategies and variety of fibers composition and structure. Additionally, 3D and 4D printing methods for scaffold production were highlighted.

In two different research articles, Tawfik et al. reported the uses of coaxial electrospun fibers for as fast dissolving matrixes. In the paper entitled “Fabrication and Characterization of Fast-Dissolving Films Containing Escitalopram/Quetiapine for the Treatment of Major Depressive Disorder” [23], authors used polyvinylpyrrolidone (PVP) as core and shell polymer for the coaxial setup to simultaneously delivery escitalopram and quetiapine, in which the first one was incorporated to the outer layer and the second on into core fiber. These fibers were fully characterized, and core-sheath structure confirmed, mainly by transmission electron microscopy. The in vitro release revealed that more than 50% of both drug molecules were released after 5 min. Moreover, using a Franz diffusion cell study, the authors showed that quetiapine permeation was enhanced compared to the control at different time points, which could be correlated to the amorphous form of the drug. In “Fast-Dissolving Nifedipine and Atorvastatin Calcium Electrospun Nanofibers as a Potential Buccal Delivery System” [24], Tawfik et al reported preparation and characterization of PVP electrospun fibers. Using the strategy for simultaneous delivery of atorvastatin (shell) and nifedipine (core), coaxial fibers presented a disintegration time smaller than 12 s in PBS (pH 7). The in vitro drug release experiments demonstrated a burst effect at the first 10 min, followed by a complete release of both drug molecules after 120 min. Additionally, both drugs permeation was significantly improve based on the incorporation into the PVP fibers comparing to the free molecules.

Chen et al. demonstrated uses of aligned core-shell fiber for the delivery of docosahexaenoic acid and brain-derived neurotropic factor (“Co-Delivery of Docosahexaenoic Acid and Brain-Derived Neurotropic Factor from Electrospun Aligned Core–Shell Fibrous Membranes in Treatment of Spinal Cord Injury”) [25]. The core-shell fibers were characterized by microscopy techniques, confirming the coaxial structure. In addition, the profile release evidenced a two-stage drug release behavior for both molecules. A burst release was followed by sustained release for up to 50 days. In vitro and in vivo experiments were also carried out. A higher than 90% cell viability was found for all systems, and the efficiency of an aligned in guiding neuronal outgrowth was evaluated. In a spinal cord injury animal model, core-shell fibrous membrane mediated drug delivery, resulting in the capacity for providing neuroprotection and promoting neuroplasticity changes in neuronal tissue.

The research article “Telmisartan Loaded Nanofibers Enhance Re-Endothelialization and Inhibit Neointimal Hyperplasia” by Lee, Kuo, Liu and co-authors demonstrated the electrospun nanofibers loaded with telmisartan for sustained and local delivery of drug to injured arterial vessels [26]. Poly(d,l)-lactide-*co*-glycolide (PLGA) nanofibers were loaded and spun on the metal stent. Electrospun fibers were characterized and the presence of drug on the material’s surface was suggested based on water contact angle. The drug release was evaluated, in which the telmisartan was released over 30 days. In vitro and in vivo results of hybrid stent/PLGA nanofibers loaded with telmisartan indicated that this material can controllably release disease-relevant therapeutics, enhance the migration of endothelial progenitor cells, providing complete endothelial coverage and recovery, and reducing intimal hyperplasia.

Snetkov et al. reported in their research article (In-Vitro Antibacterial Activity of Curcumin-Loaded Nanofibers Based on Hyaluronic Acid against Multidrug-Resistant ESKAPE Pathogens) evaluated the antibacterial activity of the fibers loaded with curcumin against polyresistant bacteria, using hyaluronic acid sodium as a polymer basis [27]. Based on the microscopy analysis, fibers diameters vary from few nanometers to 400 nm, depending on the composition. The surface properties from the nanofibers were also analyzed and the presence of curcumin enhanced the water contact angle. Physical-chemical analysis confirmed the fiber composition, and the in vitro drug release for all system evaluated. These results indicated that no burst release occurred. The antimicrobial activity data revealed the ability of curcumin-loaded nanofibers to be active against both Gram-positive and Gram-negative strains.

In terms of biologic applications, Pisani et al., in their research entitled “Engineered Full Thickness Electrospun Scaffold for Esophageal Tissue Regeneration: From In Vitro to In Vivo Approach” [28], developed a full-thickness poly-l-lactide-*co*-poly-*ε*-caprolactone (PLA-PCL) electrospun tubular scaffold engineered with mesenchymal stem cells (MSCs), in order to promote and speed up the regeneration process, ensuring an adequate support to esophageal tissue reconstruction, avoiding the use of autologous conduits. They observed an interesting correlation between the asymmetrical shape of the scaffold and its p-MSCs cellularization process, which helped in keeping the scaffold’s mechanical properties suitable for esophageal application. The authors also observed that the advantage of their pro-posed scaffold with hybrid composition (polymer combined to p-MSCs). Preliminary results collected after *in vivo* implantation in a porcine model were promising; but further studies are required to statistically validate the data.

Steinberg et al., (Novel In Situ-Cross-Linked Electrospun Gelatin/Hydroxyapatite Nonwoven Scaffolds Prove Suitable for Periodontal Tissue Engineering) [29], developed new nonwoven of 16% gelatin/5% hydroxyapatite electrospinning based scaffolds for periodontal tissue engineering. Additional porosity was incorporated via extractable polyethylene glycol fibers to allow human mesenchymal stem cells (hMSCs) and periodontal ligament fibroblasts (PDLFs) colonization and penetration. In vitro tests showed that these novel in situ crosslinked electrospun nonwoven scaffolds allowed efficient adhesion and survival of hMSCs and PDLFs. Coordinated expression of differentiation markers were also observed, which rendered this platform an interesting candidate for periodontal tissue engineering.

Kim, D., Kim, S., and co-authors detailed in their article entitled Enhanced Differentiation Capacity and Transplantation Efficacy of Insulin-Producing Cell Clusters from Human iPSCs Using Permeable Nanofibrous Microwell-Arrayed Membrane for Diabetes Treatment [30] a biodegradable polycaprolactone (PCL) electrospun nanofibrous microwell arrayed membrane permeable to soluble factors to improve some challenges related to insulin-dependent diabetes therapy. Stem cell-derived insulin producing cells (IPCs) were seeded into the electrospun microwell with a great cell survival rate. Thus, it was possible to conclude that nanofibrous microwell-arrayed membrane could be a new platform to improve IPC differentiation capacity allowing progress related to in situ transplantation technique for diabetic patients.

Dean, Thomas, and co-authors detailed, in “Uni-Directionally Oriented Fibro-Porous PLLA/Fibrin Bio-Hybrid Scaffold: Mechano-Morphological and Cell Studies” [31], a biohybrid oriented fibrous scaffold based on nanofibers of poly(l-lactic acid) (PLLA)/fibrin produced by electrospinning for ligament-replacement grafts. Mimetic of collagen bundles in size, orientation, and mechanical function is desired for ligament engineering applications. Based on the results, the authors hypothesize that a combination of fiber orientation/alignment and immobilization of fibrin can result in mechanical and morphological modification of PLLA tissue scaffolds. Further, it was found that treatment with NaOH enhanced the osteogenic differentiation of hMSCs and the additional inclusion of fibrin further enhanced osteogenic differentiation.

Currently, numerous polymers, blends and hybrid systems have been electrospun in order to improve these materials in biomedical applications. In addition to the several approaches in order to obtain electrospun fibers with a vast range of morphologies, including coaxial and side-by-side nozzles, and different collectors, combining this strategy with others microfabrication techniques has increased the applicability of these materials for different biological systems and chemical sciences.

## Figures and Tables

**Figure 1:**
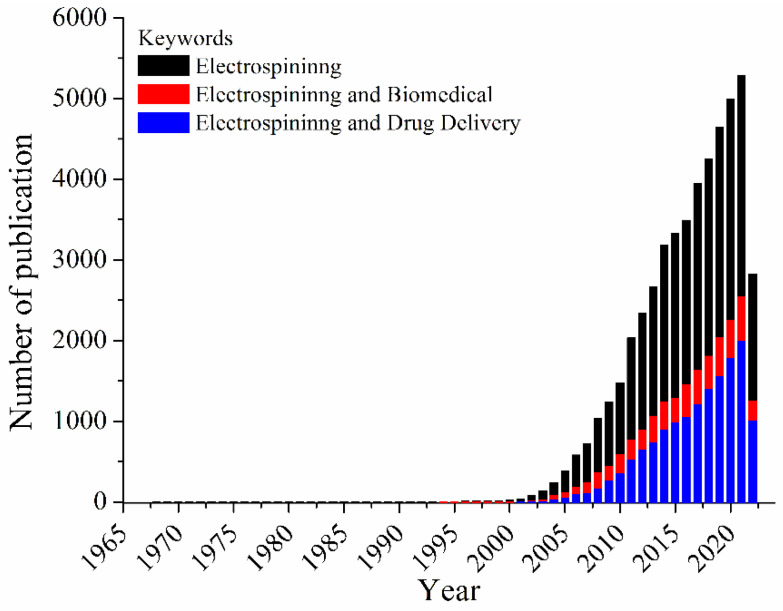
Number of documents per year, considering the keywords: electrospinning, electrospinning and biomedical or electrospinning and drug delivery.
